# Sensing of Proteins by ICD Response of Iron(II) Clathrochelates Functionalized by Carboxyalkylsulfide Groups

**DOI:** 10.3390/biom10121602

**Published:** 2020-11-26

**Authors:** Mykhaylo Losytskyy, Nina Chornenka, Serhii Vakarov, Samuel M. Meier-Menches, Christopher Gerner, Slawomir Potocki, Vladimir B. Arion, Elzbieta Gumienna-Kontecka, Yan Voloshin, Vladyslava Kovalska

**Affiliations:** 1 Institute of Molecular Biology and Genetics NASU, 150 Zabolotnogo St., 03143 Kyiv, Ukraine; v.kovalska@gmail.com; 2 Vernadsky Institute of General and Inorganic Chemistry NASU, 32/34 Palladina Av., 03142 Kyiv, Ukraine; nina.v.chornenka@gmail.com (N.C.); vakarov.s.v@gmail.com (S.V.); 3 Department of Analytical Chemistry, University of Vienna, Währinger Strasse, 38, A-1090 Vienna, Austria; samuel.meier@univie.ac.at (S.M.M.-M.); christopher.gerner@univie.ac.at (C.G.); 4 Faculty of Chemistry, University of Wroclaw, 14 F. Joliot-Curie St., 50-383 Wroclaw, Poland; slawomir.potocki@chem.uni.wroc.pl (S.P.); elzbieta.gumienna-kontecka@chem.uni.wroc.pl (E.G.-K.); 5 Department of Inorganic Chemistry, University of Vienna, Währinger Strasse, 42, A-1090 Vienna, Austria; vladimir.arion@univie.ac.at; 6 Nesmeyanov Institute of Organoelement Compounds RAS, 28 Vavilova St., 119991 Moscow, Russia; voloshin@ineos.ac.ru; 7 Kurnakov Institute of General and Inorganic Chemistry RAS, 31 Leninsky prosp., 119991 Moscow, Russia

**Keywords:** iron(II) clathrochelates, globular proteins, albumins, CD reporters, ITC, ESI-TOF MS, fluorescence quenching

## Abstract

Recognition of elements of protein tertiary structure is crucial for biotechnological and biomedical tasks; this makes the development of optical sensors for certain protein surface elements important. Herein, we demonstrated the ability of iron(II) clathrochelates (**1**–**3**) functionalized with mono-, di- and hexa-carboxyalkylsulfide to induce selective circular dichroism (CD) response upon binding to globular proteins. Thus, inherently CD-silent clathrochelates revealed selective inducing of CD spectra when binding to human serum albumin (HSA) (**1**, **2**), beta-lactoglobuline (**2**) and bovine serum albumin (BSA) (**3**). Hence, functionalization of iron(II) clathrochelates with the carboxyalkylsulfide group appears to be a promising tool for the design of CD-probes sensitive to certain surface elements of proteins tertiary structure. Additionally, interaction of **1**–**3** with proteins was also studied by isothermal titration calorimetry, protein fluorescence quenching, electrospray ionization mass spectrometry (ESI-MS) and computer simulations. Formation of both 1:1 and 1:2 assemblies of HSA with **1**–**3** was evidenced by ESI-MS. A protein fluorescence quenching study suggests that **3** binds with both BSA and HSA via the sites close to Trp residues. Molecular docking calculations indicate that for both BSA and HSA, binding of **3** to Site I and to an “additional site” is more favorable energetically than binding to Site II.

## 1. Introduction

Proteins are the main working instruments of a living organism. These macromolecules are precise tools for performing various biological functions, such as fermentative catalysis, transport and sensing [1,2]. Most proteins manifest these properties by using the unique tertiary structure of their polypeptide chains, forming the surface with specific location of amino acid residues, which are able to form hydrogen bonds, salt bridges or hydrophobic domains [2,3,4]. This unique structure defines the possibility of interactions with complimentary ligands or protein/nucleic acids motifs, and is thus responsible for specific functions of protein macromolecule [5,6,7]. On the other hand, the changes in a protein’s tertiary structure (in particular, due to the so-called “misfolding”) can affect the functions of a given protein, or even make it toxic to a living organism (such as in the case of prions [8,9,10,11] or amyloid fibrils [12,13,14,15,16]). At the same time, changes in a protein’s tertiary structure (e.g., misfolding) lead to the change in the layout of the protein amino acid residues able to contact with medium or ligands (we will call them “surface residues”, even if they are in the protein binding site) [17,18,19]. Therefore, it seems to be undoubtedly important to be able to detect, observe and describe certain surface elements (i.e., areas of adjacent protein surface residues, particularly those able to bind some molecules) of folded (or misfolded) protein macromolecules and also to monitor the changes in these elements at various levels [20,21,22], as these changes would reflect the overall protein conformation changes or misfolding. The development of convenient and reliable (preferably optical) molecular sensors/probes, which are able to bind selectively to the above surface elements, thus giving a specific and clear response on this supramolecular assembly, is highly required.

Metal clathrochelates are the three-dimensional cage complexes with an encapsulated metal ion [23,24]. The size and volume of their macropolycyclic structures are substantially larger than those of the regular low-molecular-weight (mainly organic) probes. The 3D-geometry of these molecules and the ability of their easy modification by using a wide range of various functionalizing groups in the apical and ribbed positions make them prospective molecular scaffolds for the design of molecular sensors, which are able to selectively bind to the target elements of a protein’s surface.

Recently, it has been discovered that the binding to bovine serum albumin (BSA) makes the optically inactive iron(II) clathrochelates functionalized with carboxyphenylsulfide groups able to induce circular dichroism spectra (ICD) [25]. As an explanation to this phenomenon, we suggested that BSA binding sites are suited to predominant fixation of one of the two (Λ or Δ) trigonal prismatically (TP)—trigonal antiprismatically (TAP)—distorted conformations of the clathrochelate framework [26]. The iron(II) clathrochelate isomers with six carboxyphenylsulfide groups were reported to give a substantially different ICD response upon binding to BSA and to human serum albumin (HSA), thus allowing us to discriminate between these two structurally related proteins [26]. In addition, the clathrochelates with two carboxyphenylsulfide substituents have shown different optical responses upon their supramolecular assembly with globular proteins, permitting to distinguish between albumins and beta-lactoglobulin (BLG) [24]. Since CD spectra are known to be sensitive to geometrical distortions of the absorbing chromophores, the ability of the clathrochelate framework to adopt left or right TP–TAP twisted conformations makes these cage metal complexes suitable for the detection of the certain elements of protein tertiary structure and discerning between these elements [24]. It should also be mentioned that the intensities and shapes of the clathrochelate CD spectra induced by the presence of the proteins are substantially affected by the constitutional isomerism of the clathrochelates with carboxyphenylsulfide ribbed substituent—i.e., the ortho-, meta- or para-position(s) of their terminal carboxyl group(s) [26,27]. Moreover, the data of isothermal titration calorimetry (ITC) suggest that the clathrochelates-to-protein binding stoichiometry is also affected by this type of isomerism being equal to 1:2 for the para-substituted isomer and to 1:1 for its ortho- and meta-substituted analogs [26].

However, a very low-intensity ICD response of the iron(II) clathrochelate with two carboxyalkylsulfide substituents to the presence of albumin (BSA) was previously reported [25], while its binding led to a substantial quenching of BSA protein fluorescence, thus implying the formation of supramolecular assembly. Therefore, the study of the ability of clathrochelate analogs with a different number of the carboxyalkylsulfide groups to induce the CD response on their binding with various proteins is of a great interest.

**Scheme 1:**
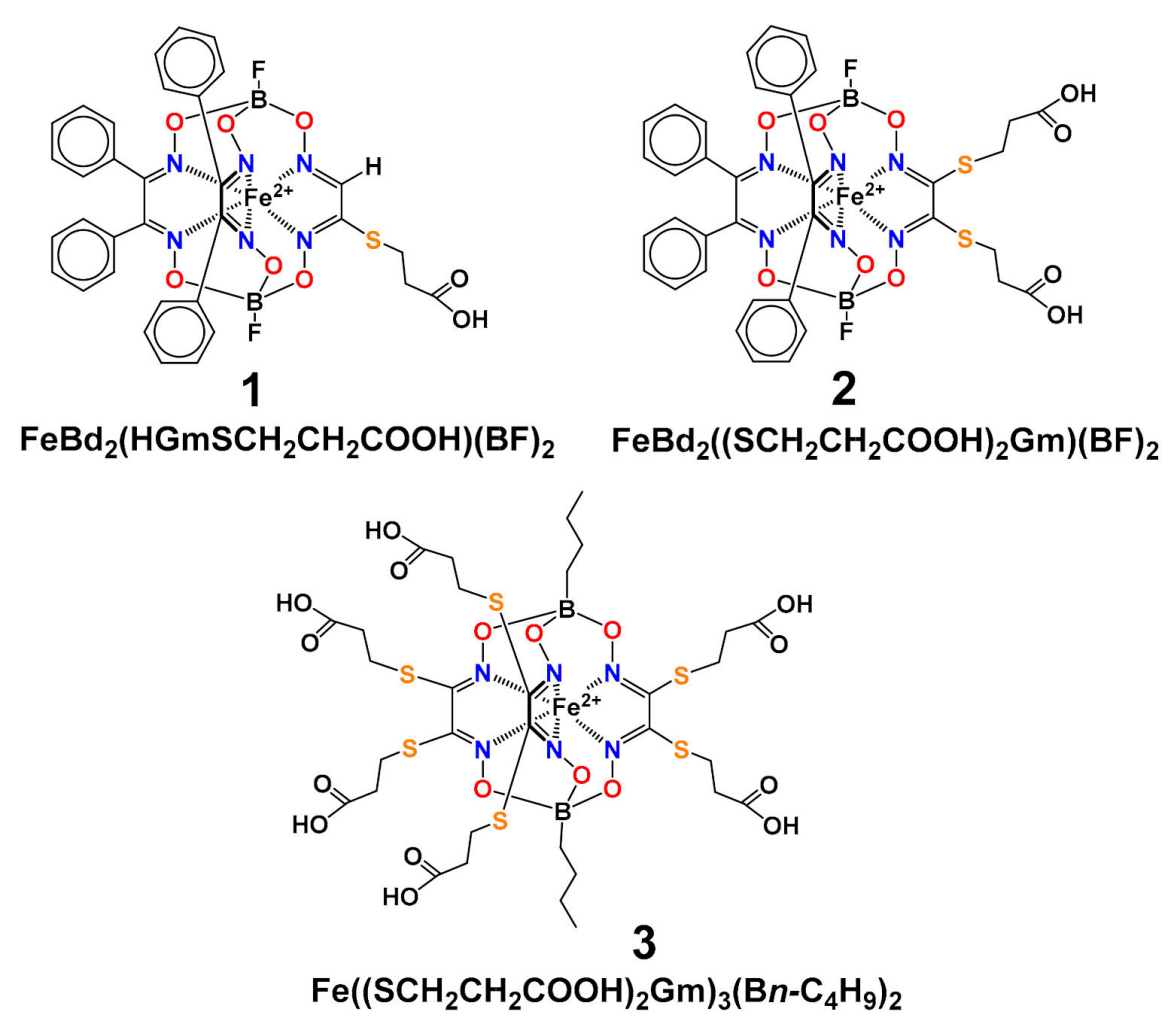
Chemical drawings of the carboxyl-terminated alkylsulfide iron(II) clathrochelates **1**–**3**.

This work is aimed at the study of the ability of iron(II) clathrochelates functionalized with “flexible” alkylcarboxy substituents (Scheme 1) for ICD sensing of globular proteins, as well as the dependence of this ability on the quantity of alkylcarboxy substituents, and the peculiarities of clathrochelates binding to protein globules. Thus, herein, we report the results of our studies of interactions between globular proteins (BSA, HSA, BLG and lysozyme (LYZ)) with mono-, di- and hexa-carboxyalkylsulfide functionalized iron(II) clathrochelates and exploration of clathrochelate’s CD sensitivity to these proteins. In addition to the CD spectroscopy, ITC and fluorescent spectroscopy were used to characterize the clathrochelate—protein supramolecular assemblies. Electrospray ionization mass spectrometry (ESI-MS) was successfully applied for experimental detection of these assemblies under native conditions. The computer simulations of the non-covalent assemblies of the clathrochelates with BSA and HSA macromolecules were also performed, and the results discussed.

## 2. Materials and Methods

### 2.1. Materials

BSA and HSA were obtained commercially [SAF^®^ (St. Louis, MI, USA) and Fisher Bioreagents (Waltham, MA, USA), respectively). BLG, LYZ and DMSO of an analytical grade were also purchased from SAF^®^. Tris–HCl aqueous buffer (50 mM) with pH 7.9 was used as a solvent in all the spectral experiments.

The dicarboxyalkylsulfide iron(II) clathrochelate (**2**) was prepared as described elsewhere [25]. The clathrochelate precursors FeBd_2_(HClGm)(BF)_2_ and Fe(Cl_2_Gm)_3_(BF)_2_ were obtained as described previously [28,29]. Monocarboxyalkylsulfide iron(II) clathrochelate (**1**) and hexacarboxyalkylsulfide iron(II) clathrochelate (**3**) were synthesized as described below.

FeBd_2_(HGmSCH_2_CH_2_COOH)(BF)_2_ (clathrochelate **1**). A solution of FeBd_2_(HClGm)(BF)_2_ (0.7 g, 1.0 mmol), mercaptopropionic acid (0.13 g, 1.2 mmol) and triethylamine (0.35 mL, 2.5 mmol) in dry dichloromethane (100 mL) was stirred at room temperature for 5 h. The reaction course was controlled via TLC on silica gel by using dichloromethane as eluent. Then the reaction mixture was washed with 0.1% aqueous hydrochloric acid (500 mL) and water (500 mL), and the dichloromethane extract was evaporated to dryness. The crude solid was separated by column chromatography on silica gel (eluent: dichloromethane–*iso*-propylamine 99:1). The major elute was collected and evaporated to dryness. The solid residue was extracted with dichloromethane and the product was precipitated with hexane. The compound was filtered off, washed with hexane and dried in vacuo. Yield: 0.70 g, 88%. ^1^H NMR (DMSO-d_6_), δ, ppm: 1.9 (d, 2H, CH_2_), 4.32 (d, 2H, CH_2_), 7.31 (m, 20H, Ph), 8,54 (s, 1H). ^13^C{^1^H} NMR (DMSO-d_6_) δ, ppm: 32.86 (s, CH_2_), 40.15 (s, CH_2_), 125.51, 128.00, 128.81, 128.84, 130.12, 130.43, 130.52(s, Ph), 156.14(s, SC=N), 156.61(s, HC=N), 169.90 (s, COOH). MS m/z: 782.11 [M].

Fe((HOOC(CH_2_)_2_S)_2_Gm)_3_(Bn-C_4_H_9_)_2_ (clathrochelate **3**). A solution of Fe(Cl_2_Gm)_3_(Bn-C_4_H_9_)_2_ (0.65 g, 1.0 mmol), mercaptopropionic acid (0.75 g, 7.0 mmol) and triethylamine (2 mL, 15.0 mmol) in dry DMSO (5 mL) was stirred at room temperature for 2 h, and then additional mercaptopropionic acid (0.21 g, 2.0 mmol) and triethylamine (0.55 mL, 4.0 mmol) were added. The reaction course was monitored via TLC on silica gel with dichloromethane as eluent. The reaction mixture was diluted with water, extracted with dichloromethane (300 mL). Organic layer was washed with 0.1% aqueous hydrochloric acid (500 mL) and water (500 mL). The dichloromethane phase was evaporated to dryness and the solid residue was separated by column chromatography on silica gel (eluent: dichloromethane–*iso*-propylamine 99:1). The major elute was collected and evaporated to dryness. The solid residue was extracted with dichloromethane and the extract was precipitated with hexane. The product was filtered off, washed with hexane and dried in vacuo. Yield: 0.4 g, 37%. ^1^H NMR (DMSO-d_6_), δ, ppm: 0.6 (s, CH_3_), 0.89 (s, CH_2_-**CH_2_**-CH_3_), 1.05 (s, S-**CH_2_**-CH_2_), 1.26 (s, B-**CH_2_**-CH_2_), 1.38 (s, CH_2_-**CH_2_**-COOH), 146.89(s, SC = N), 171.80 (s, COOH). ^13^C{^1^H} NMR (DMSO) δ, ppm: 14.02 (s, CH_3_), 25.30 (s, CH_2_), 26.17 (s, CH_2_), 29.02 (s, CH_2_), 34.43 (s, CH_2_), 146.89(s, SC=N), 171.80 (s, COOH). MS *m*/*z*: 1073.00 [M].

### 2.2. Circular Dichroism Studies

CD spectra were recorded on a Jasco J-1500 spectropolarimeter at room temperature in the range 300–600 nm; three scans were averaged for each of these ICD spectra. The data are expressed as ellipticity (mdeg), obtained in mdeg directly from the instrument. Tris–HCl aqueous buffer with pH 7.9 was used for preparation of the protein’s stock solutions, as well as of the samples with a protein-to-clathrochelate 2:1 molar ratio (*c_protein_* = 4 × 10^–5^ M, *c_clt_* = 2 × 10^–5^ M).

### 2.3. ITC Experiments

Working solutions with the clathrochelate-to-protein molar ratios from 0.25:1 to 3.6:1 were prepared by mixing of 6 mM solution of the clathrochelate **3** in 0.1 M aqueous sodium phosphate buffer and 0.5 mM BSA solution in 0.1 M aqueous sodium phosphate buffer. It should be mentioned that from the studied clathrochelates, only **3** possessed the solubility in water appropriate for the conditions of ITC experiment. ITC experiments were performed using a Nano ITC calorimeter (TA Instruments, New Castle, DE, USA) equipped with a standard 1.0-mL-in-volume cell (24K Gold). A stock solution of the clathrochelate **3** was added to this cell using a 250-μL syringe. The calorimetric experiments were operated using the Nano ITC Run v. 2.2.3 software. All the experimental data were evaluated using the NanoAnalyze v. 2.4.1 program package and an independent model was used for their interpretation. In each of the cases, the corresponding control experiment was performed and enthalpies of a dilution of its components were subtracted from the experimental data obtained for their mixture.

### 2.4. Protein Fluorescence Quenching Studies

To study in more detail the interaction of one of the clarhtochelates with HSA and BSA, the quenching of these proteins’ fluorescence with clathrochelate **3** was performed (this clathrochelate was chosen for the detailed study, since unlike the two others, it could be studied by ITC). The UV-vis absorption spectra of BSA and HSA in the absence and in the presence of the clathrochelate **3** were recorded using a Shimadzu UV-3600 UV-VIS-NIR spectrophotometer. Their fluorescence spectra were measured on a Cary Eclipse fluorescence spectrophotometer (Varian, Palo Alto, CA, USA). Tris–HCl aqueous buffer (50 mM) with pH 7.9 was used for preparation of the solutions of these albumins with *c_protein_* = 3 × 10^–6^ M; an aliquot of the freshly prepared 2 mM DMSO solution of the clathrochelate **3** was added to the obtained protein’s solution; the concentration of **3** was varied from 10^–6^ M to 15 × 10^–6^ M. Fluorescence spectra of the above solutions were measured using an excitation at 295 nm to avoid the excitation of the tyrosine residues of BSA and HSA macromolecules. Titrations of BSA and HSA with **3** were repeated three times and the corresponding average values were evaluated. Even though the amount of the added aliquots of the DMSO solution of **3** was rather small (less than 1% of the total volume), DMSO was found to slightly affect the protein fluorescence intensity. To estimate its effects, the same aliquots of DMSO were added to the initial solutions of BSA and HSA albumins, and the corresponding changes in their protein fluorescence intensities were measured; these changes were taken into account in the case of the samples containing corresponding aliquots of clathrochelate **3**. Additionally, an addition of clathrochelate **3** caused an increase in the optical densities of BSA/HSA protein-containing solutions at wavelengths of fluorescence excitation and emission, thus decreasing their intensity due to the so-called “inner filter effect” (IFE) and the reabsorption process. To avoid the errors caused by these factors, the intensities of the protein fluorescence spectra in the presence of cage complex **3** were corrected using Equation (1):

(1) $$I_{cor}=\;I_{obs}\times 10^{\;\frac{D_{ex}+\;D_{em}}{2}},$$

where I_cor_ and I_obs_ are the corrected and observed fluorescence intensities, respectively; D_ex_ and D_em_ are the clathrochelate’s optical densities at the wavelengths of the protein’s excitation and emission, respectively.

The binding constants and stoichiometry of the corresponding clathrochelate–protein assemblies were estimated for BSA and HSA as the hosts and the clathrochelate **3** as the guest. This estimation was performed under a suggestion that each protein globule has *n* sites for clathrochelate binding with the equal values of the binding constant *K*. Another suggested condition was that the binding of clathrochelate to each binding site causes the same degree of quenching of the protein tryptophan fluorescence. To obtain the values of *K* and *n*, the experimentally obtained dependences of a protein fluorescence quenching were presented as the plots of (*1 − I/I_0_*) (where *I_0_* and *I* are the protein fluorescence intensities in the absence and presence of clathrochelate **3**) versus the concentration of **3** (*c_clt_*). Then, they were fitted by using Equation (2) (which was obtained as described in [27]):

(2) $$Y=A\;\times \;\left[\frac{1}{2}+\;\frac{x}{2\;\times c_{P}\;\times \;n}+\;\frac{1}{2\;\times K\;\times c_{P}\;\times \;n}\;-\;\sqrt{{\left(\frac{1}{2}+\;\;\frac{x}{2\;\times c_{P}\;\times \;n}+\;\frac{1}{2\;\times K\;\times c_{P}\;\times \;n}\right)}^{2}\;\frac{x}{c_{P}\;\times \;n}}\right],$$

where X = *c_clt_*; Y = 1 − *I/I_0_*; A = (1 − *I_min_/I*_0_), where *I_min_* is the protein fluorescence intensity upon occupation of all the binding sites of a given protein macromolecule by the guest clathrochelate molecules, and *c_P_* is the concentration of protein globules.

As a result of the above evaluations, the values of *K*, *n* and *A* were obtained as the fitting parameters; *K* and *A* values were also calculated with the fixed *n* values (1 or 2).

### 2.5. Electrospray Ionization Mass Spectrometry

A weighted amount of the HSA (delipidated, Sigma, St. Louis, MI, USA) was dissolved in 20 mM ammonium bicarbonate aqueous buffer to obtain a solution with concentration equal to 200 µM. A weighted amount of a given cage iron(II) complex was dissolved in methanol just prior to the corresponding MS experiment to obtain a 2 mM solution. The reaction mixture containing 20 μM of the above protein and 60 μM of the clathrochelate in 20 mM aqueous ammonium bicarbonate was incubated at room temperature for 15 min.

Electrospray ionization mass spectra were measured on a Maxis QToF (Bruker Daltonics, Germany) using the Q-Tof control (Version 3.2.31.0) and the DataAnalysis (Version 4.1.359.0) software packages. The samples were injected under native conditions into the mass-spectrometer using the following parameters: infusion rate 350 μL/min, capillary voltage 4 kV, end plate offset −500 V, dry temperature 210 °C, dry gas 6 L/min, nebulizer 0.8 bar, transfer time 120 μs, acquisition rate 1 Hz and rolling average 2. The instrument was tuned to the high-mass range. Signals of HSA were detected in the range of *m/z* 3000–5000. The mass spectra were acquired over 5 min and averaged. Then, each of these experimental spectra was smoothed using the Savitzky–Golay algorithm with 0.2 smoothing width in five cycles.

### 2.6. Computer Modelling

Molecular geometry of the hexacarboxyethylsulfide iron(II) clathrochelate **3** was calculated by consecutive optimization: in the first stage, with the MOPAC 2016 program [30], PM7 level [31] and with the ORCA 4.2.0 package [32,33] (the PBE [34] functional (def2-TZVP basis set [35,36]) with a dispersion correction [37]) in the next stage. Gasteiger charges were added by AutoDock Tools and then non-polar hydrogen atoms of **3** were merged and its rotatable bonds were defined. Boron atoms were manually changed to Carbon ones in pdbqt file. The optimal calculation sizes for evaluation of the molecule **3** were calculated according to [38] as the cube with a length of its edge of 27 Å; we used slightly larger grid boxes (30 × 30 × 40 Å), thus aiming to fully include its binding sites.

XRD structures of HSA monomer and BSA dimer (PDB ID: 4L8U, 4JK4) were obtained from the Protein Data Bank [39,40]. Water molecules, ligands and second macromolecule of BSA were removed. All hydrogens were added and Gasteiger charges computed. Then, non-polar hydrogens were merged. The docking calculations were performed using the AutoDock Vina program (1.1.2, The Scripps Research Institute, La Jolla, CA., USA) and the MGLTools (1.5.6, The Scripps Research InstituteManufacturer, La Jolla, CA., USA) [41,42].

**Figure 1:**
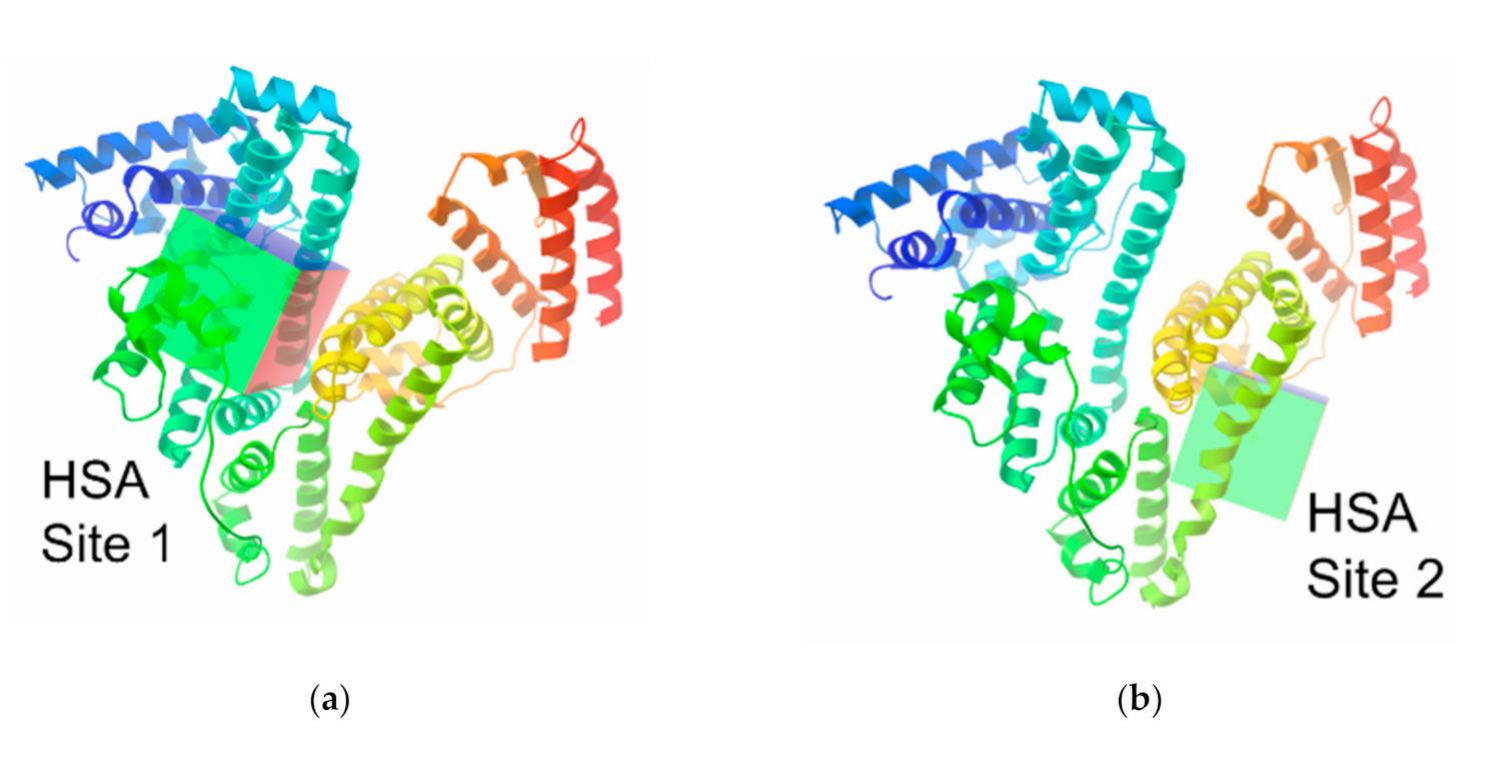

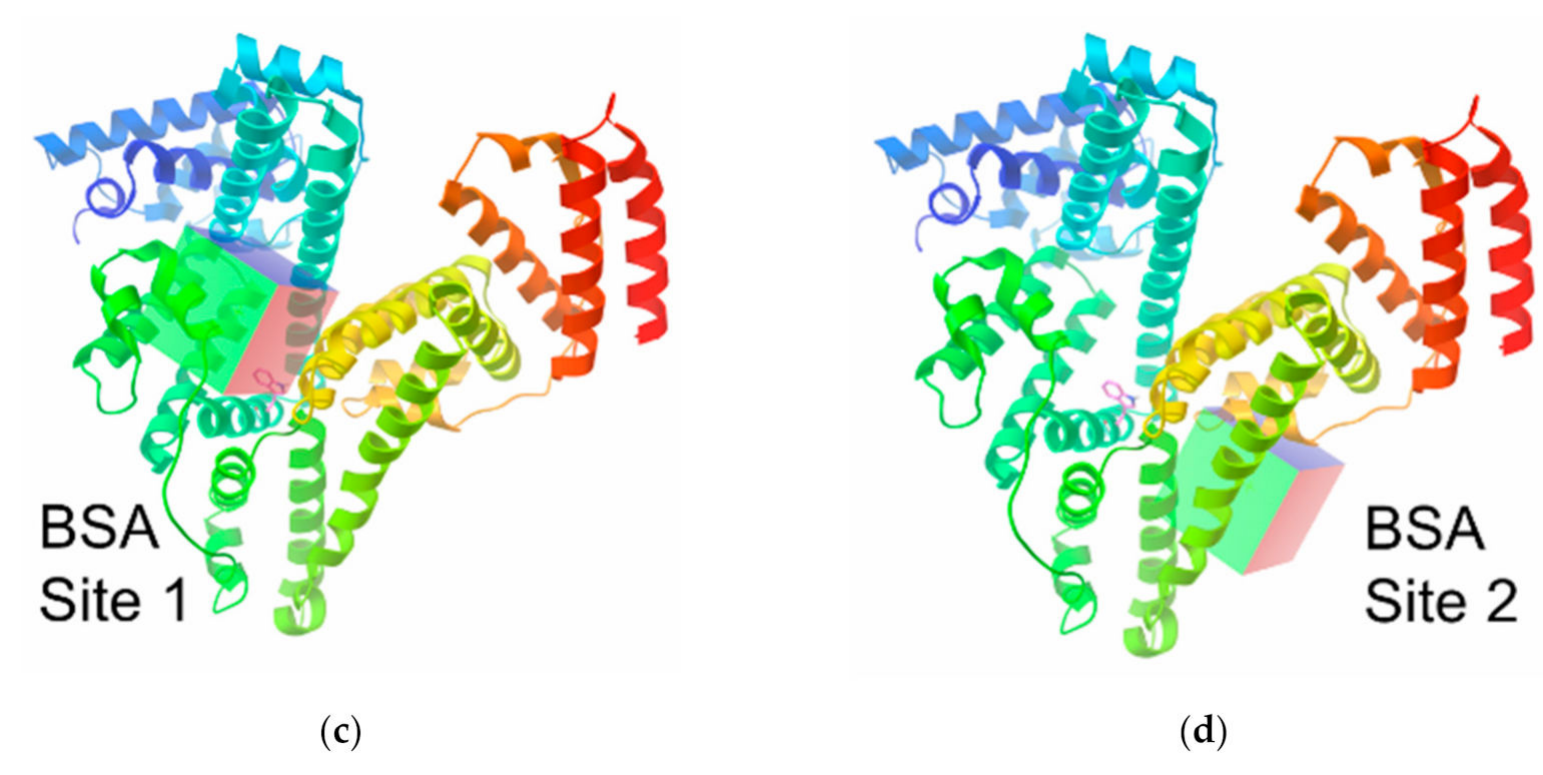
The binding sites I (**a**,**c**) and II (**b**,**d**) of human (HSA; (**a**,**b**)) and bovine (BSA; (**c**,**d**)) serum albumin macromolecule.

When exploring the entire surface of macromolecules, the centers of the macromolecules were chosen and the sizes of grid boxes were enhanced up to *x* = 120, *y* = 120 and *z* = 120 Å; the value of exhaustiveness in all of the calculations was set to 200. In the course of a computer examination of the protein binding sites (Figure 1), their coordinates (Table 1) were defined as the corresponding centers in a coordinate system of the original PDB file from the RSCB protein data bank with the above grid box size of 30 × 30 × 40 Å. For a Vina docking, the default parameters were used; the best-scoring poses, judged by a Vina docking score, were chosen and then visually analyzed by using the MGLTools. For the comparison with the results obtained by docking in AutoDock Vina, we also performed redocking in AutoDock 4.2.

## 3. Results and Discussion

### 3.1. ICD Spectra

**Figure 2:**
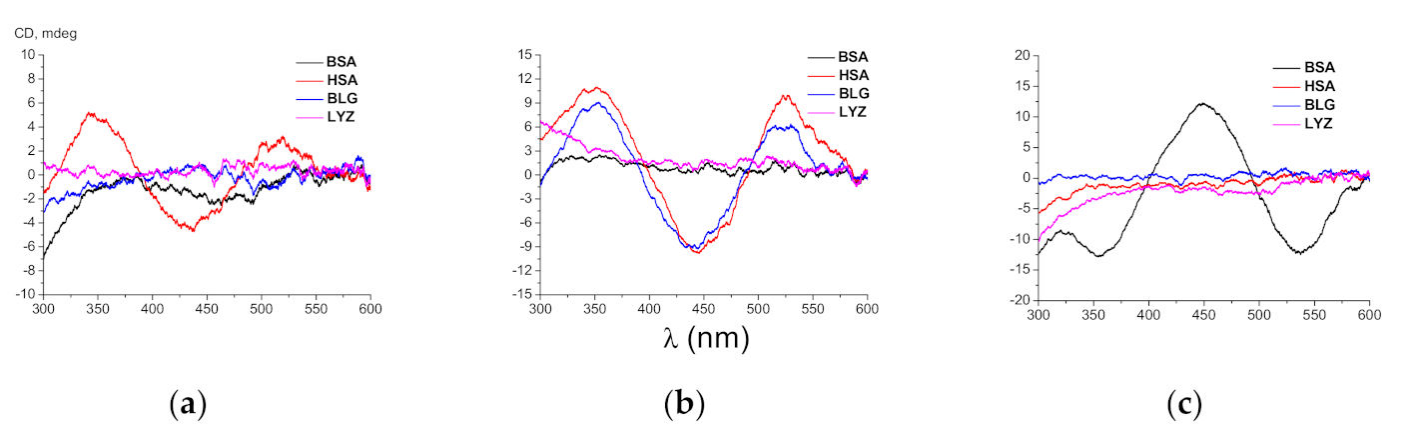
Circular dichroism (CD) spectra of the 2 × 10^–5^ M solutions of the clathrochelates **1** (**a**), **2** (**b**), and **3** (**c**) in the presence of 4 × 10^–5^ M of bovine (BSA) and human (HSA) serum albumin, lysozyme (LYZ) and beta-lactoglobulin (BLG) in 0.05 M Tris–HCl buffer with pH 7.9.

Recently, we reported on the induction of strong CD response of clathrochelates functionalized with two “structurally rigid” carboxyphenylsulfide substituents upon their binding to proteins [27]. Even though their analogue with two “structurally flexile” carboxyalkylsulfide groups exhibited a very weak ICD signal in the presence of BSA, it still strongly quenched the protein fluorescence [25]. Therefore, we examined the induction of the CD signal caused by a supramolecular binding of the iron(II) clathrochelates with one, two or six flexible carboxyalkylsulfide substituent(s) to globular proteins, such as BSA, HSA, LYZ and BLG (Figure 2).

The intensities of induced CD signals were characterized as a sum of the modules of maximum intensities of the positive and negative bands (ΔICD) in the wavelength range of 300–600 nm (Table 2).

As seen from Figure 2, clathrochelates **1**–**3** are inherently CD-silent (as it was expected), whereas the intensities and the shapes of their ICD spectra in the presence of proteins are strongly affected by both the number of the carboxyalkylsulfide group(s) per clathrochelate molecule (one, two or six) and by the nature of the hosting globular protein. In particular, the binding of **1** to HSA (Figure 2a) and **2** to HSA and to BLG (Figure 2b) resulted in the appearance of intensive ICD spectra (with ΔICDs equal to 9.8, 20.8 and 18.5 mdeg, respectively) of similar shapes with two positive (at approximately 350 and 520 nm) and one negative (at approximately 450 nm) bands. On the other hand, only weak ICD signals (with ΔICDs close to 3.0 and 2.5 mdeg, respectively) were detected for **1** and **2** in the presence of BSA. In contrast, the hexafunctionalized clathrochelate **3** in the presence of this protein gave a strong (ΔICD = 24.9 mdeg) ICD response of an inverted shape (one positive band with the maximum at approximately 450 nm and two negative bands with maxima at approximately 350 and 530 nm). At the same time, only weak ICD signals (with ΔICDs equal to 0.9 and to 0.5 mdeg, respectively) were detected for clathrochelate **3** in the presence of HSA and BLG (Figure 2c). It is worth noting that none of the three clathrochelates induced ICD signals in the presence of LYZ (with the corresponding ΔICD values in the range 0.8–1.1 mdeg).

The hexa-substituted carboxyphenylsulfide iron(II) clathrochelates were recently reported to discriminate between the structurally related serum albumins BSA and HSA by induction of CD spectra of different shapes in the presence of these proteins [26], while in the case of di-substituted macrobicyclic analogs, such discrimination has not been observed [27]. Intriguingly, each of the three clathrochelates studied in this work exhibited strong ICD response only in the presence of one of two albumins (BSA or HSA), despite their structural similarity (Figure 2). Taking into account the inverted shape of the albumin-induced CD spectra of these macrobicyclic homologs, we suggest that their high sensitivity is associated with the arrangement of binding sites of these proteins. Therefore, the number of carboxyalkylsulfide groups in ribbed positions per clathrochelate molecule strongly affects the spatial arrangement of their supramolecular complexes with a given protein macromolecule.

### 3.2. Native ESI-MS

**Figure 3:**
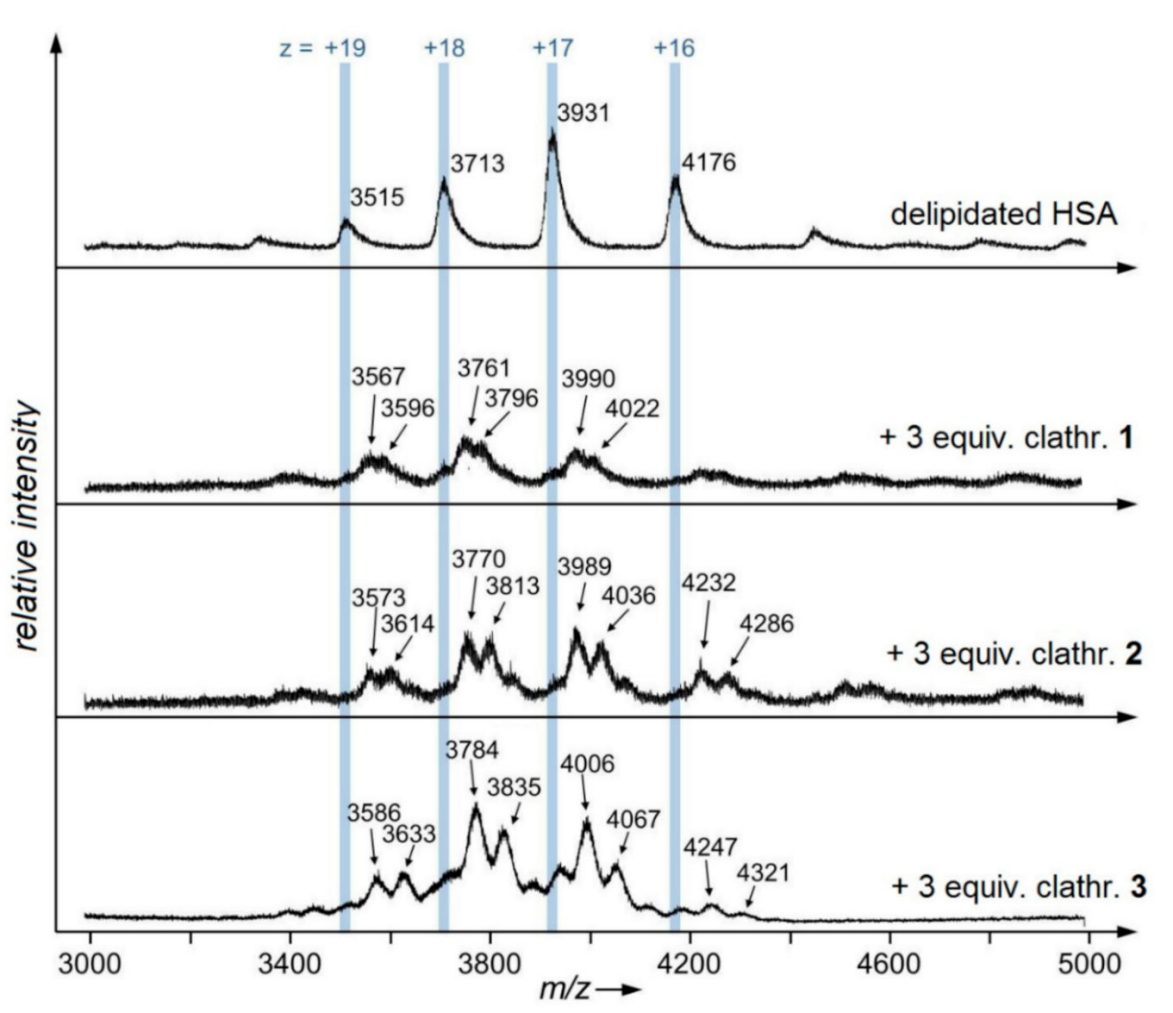
Native electrospray ionization (ESI) mass spectrum of the human serum albumin (HSA; delipidated) at its concentration of 20 µM in the absence and in the presence of the clathrochelates **1**–**3** at their concentration of 60 µM measured in 20 mM ammonium bicarbonate aqueous buffer.

ESI-MS is widely used to investigate metal–biomolecule interactions [43]. Here, the formation of the non-covalent interactions between HSA (66 kDa) and the clathrochelates **1**–**3** (about 1 kDa) was assessed using ESI time-of-flight (TOF) mass spectrometry (Figure 3). Mass spectra were recorded under native conditions that preserve the globular tertiary structure of the protein. Delipidated HSA was employed for these experiments because the associated mass signals revealed an improvement in the peak broadening compared to other sources of HSA. Averaged deconvoluted masses of the detected charge states (+15 to +19) of HSA and its adducts with the above-mentioned clathrochelates are provided in Table 3. ESI mass spectra of unreacted HSA displayed narrow peaks with a molecular mass of 66,802 ± 21 Da. Adducts of HSA with the cage complexes **1**–**3** caused a clear appearance of new signals at the expense of an unreacted albumin, which could be assigned to their protein–clathrochelate 1:1 and 1:2 adducts (Figure 3, Table 3). These signals are better resolved in the case of the polycarboxyl-terminated clathrochelates (**2** or **3**), as compared with that for monofunctionalized clathrochelate **1**. It should be noted that adducts with 1:2 HSA-to-clathrochelate stoichiometry for compounds **1** (+2 × 763 Da) and **2** (+2 × 885 Da) correspond closely to the inclusion of two discrete cage complex molecules into HSA. The larger difference between experimentally obtained and theoretically calculated values in the case of its 1:2 assembly with hexacarboxyl-terminated cage complex **3** (experimental +2 × 1142 vs. theoretical +2 × 1075 Da, respectively) suggests the inclusion of additional solvent molecules or the fragments of the macrobicyclic molecule **3**.

The performed ESI-TOF-MS experiments suggest the formation of the non-covalent adducts (i.e., supramolecular assemblies) of HSA with all these three clathrochelate homologs, while the ICD outputs in the presence of this albumin were observed only in the case of the mono- and difunctionalized clathrochelates **1** and **2** (their hexafunctionalized analog remained CD-silent). As it arises from the ESI-TOF data, all of them are able to form the supramolecular 1:1 and 1:2 protein-to-clathrochelate associates with HSA.

### 3.3. Protein Fluorescence Quenching Data

**Figure 4:**
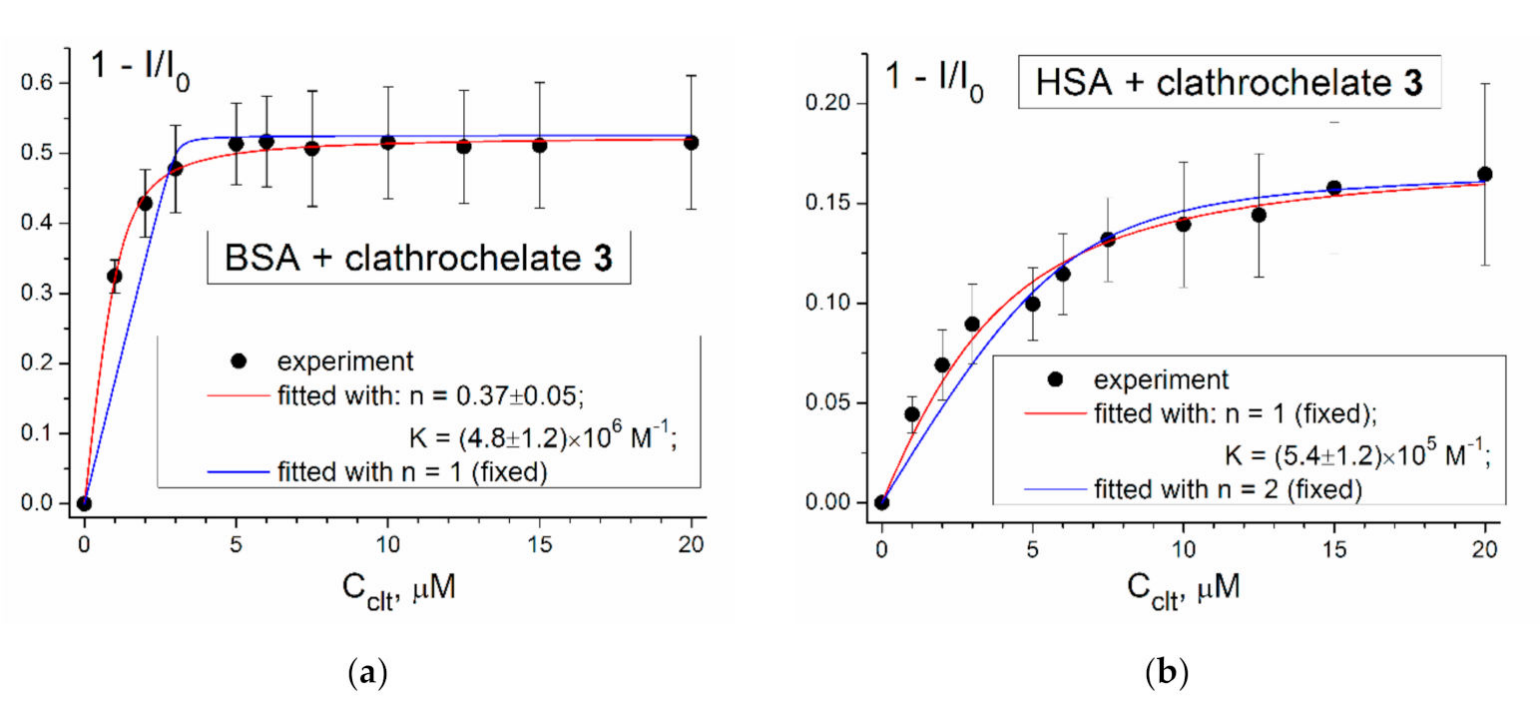
The plots of (1 *− I/I*_0_) vs. the concentration of the clathrochelate (C_clt_) for a titration of 3 µM of bovine (BSA; (**a**)) and human (HSA; (**b**)) serum albumin with the clathrochelate **3**, and the fit of these plots with the equation (2); I and I_0_ are protein fluorescence intensities in the presence and in the absence of **3** respectively. Tris–HCl buffer (0.05 M) with pH 7.9 was used as a solvent. Experiment performed three times, average values of (1 *− I/I*_0_) are presented; error bars correspond to standard deviation of the mean with confidence level of 0.9. Binding constant (K) value is found as the parameter of fitting; number of binding sites per globule (n) value is fixed where mentioned (see the corresponding legends) or found as the parameter of fitting otherwise.

Supramolecular assembling of the studied albumins with clathrochelate **3** was also studied using quenching of their protein fluorescence. BSA and HSA are known to contain two (Trp-134 and Trp-213) and one (Trp-214) fluorescent tryptophan residues, respectively; to exclude the contribution of their tyrosine residues, a protein’s emission was excited at 295 nm. The plots of the protein’s fluorescence intensity (represented in the coordinates 1 − *I/I*_0_) versus the concentration of the macrobicyclic clathrochelate **3** are shown in Figure 4.

As can be seen from Figure 4, titration of BSA and HSA with a cage complex **3** caused the quenching of the protein fluorescence; upon saturation, the corresponding emission intensities went down by approximately 50% and 20%, respectively. These results provide further evidence for the binding of **3** to both the BSA and HSA macromolecules, and indicate that the binding affects their tryptophan residues. The values of the corresponding stability constants (K) and stoichiometry (n) of these clathrochelate-to-protein adducts were estimated; the results of the fitting were different for the two albumins. In the case of BSA, the best fitting parameters were obtained at *n* = 0.37, whereas those at the fixed value of *n* = 1 were unsatisfactory (Figure 4a). Bad consistency of the used model for the BSA, containing two tryptophan residues, could explain such fitting results. In the case of existence of several (more than one) binding sites, binding into different sites would cause different degrees of quenching of Trp fluorescence, and this could be responsible for the poor fitting of the titration curve.

In the case of HSA, fitting of the corresponding quenching plot gave no reasonable value of *n* as an approximation parameter. On the other hand, those performed at the fixed *n* values (*n* = 1 or 2) suggest that at *n* = 1 the fitting quality is quite good, whereas at *n* = 2 it is less appropriate (Figure 4b). Thus, the obtained fluorescent titration data suggest the formation of 1:1 clathrochelate **3**–HSA supramolecular assembly. At the same time, the ESI-TOF mass spectrometry data evidenced the formation of both 1:1 and 2:1 complex **3**–HSA associates. This disagreement may be explained if we suppose that only one of the two clathrochelate guest molecules upon their binding with HSA affects its buried Trp-214 residue. It should be noted that the hexacarboxyalkylsulfide iron(II) cage complex **3** caused a substantially (approximately three-fold) lower quenching of BSA protein fluorescence, as compared with its earlier-described hexacarboxyphenylsulfide iron(II) clathrochelate counterpart [26]. Stronger quenching in the latter case may be attributed to the possibility of π-stacking interactions of aromatic moieties of the carboxyphenylsulfide substituents of the clathrochelate molecules with aromatic Trp residues of a hosting protein macromolecule.

### 3.4. Thermodynamic Characteristics of the Supramolecular Clathrochelate **3**–BSA Assembly

**Figure 5:**
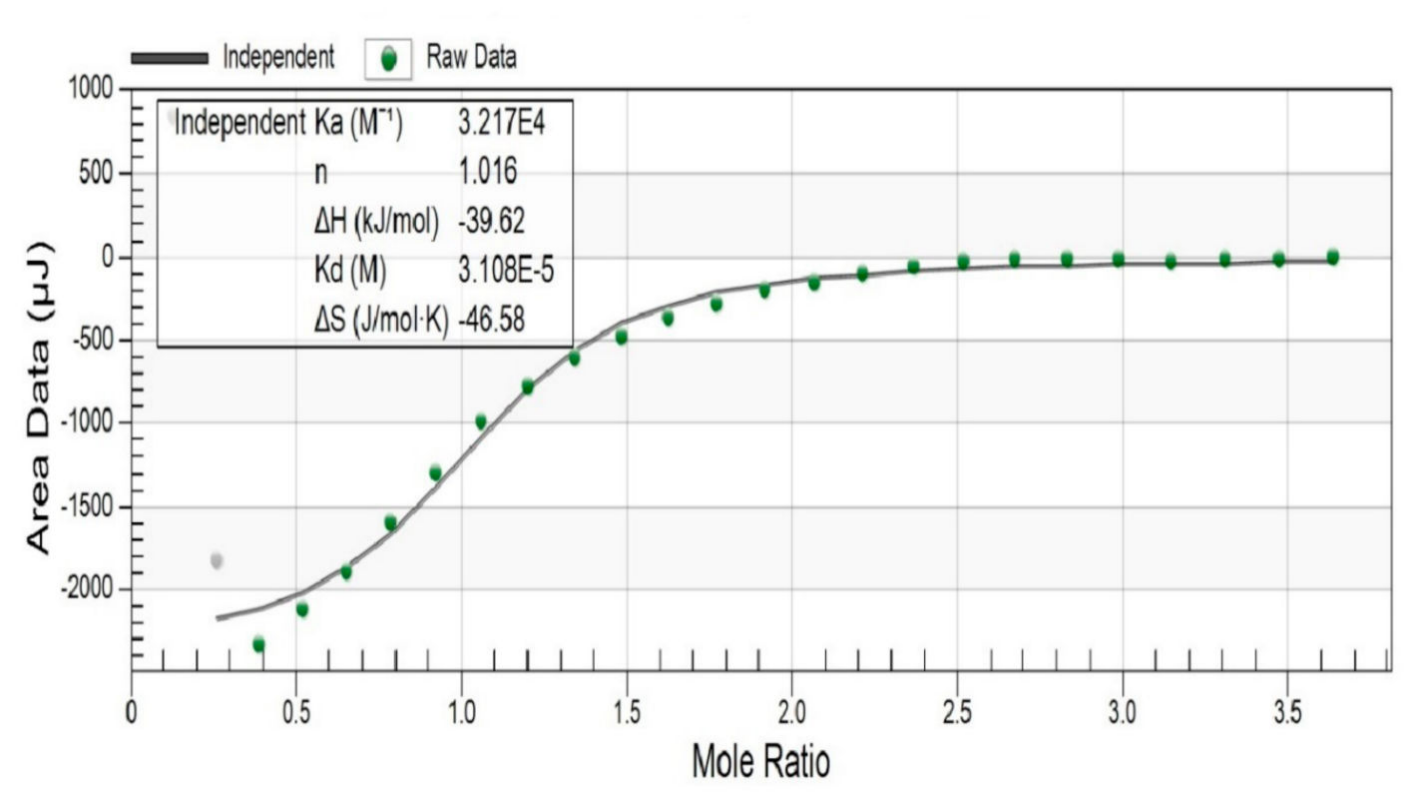
The experimental (depicted in the points) and fitted (depicted in the solid lines) plots of the binding heat effect versus the clathrochelate **3**-to-BSA (bovine serum albumin) molar ratio.

In order to gain more insight into the binding of the clathrochelate **3** to BSA, the thermodynamic parameters of this supramolecular interaction were determined by the ITC technique (Figure 5). The results of calorimetric study suggest the formation of 1:1 assembly of BSA with clathrochelate **3**; the equilibrium constant of this association K_a_ = 3.2 × 10^4^ M^–1^ (that corresponds to Gibbs free energy of ΔG = −25.7 kJ mol^–1^). Performed data fitting and calculations of the binding isotherm suggest a strongly enthalpy-driven binding mechanism (Δ*H* = −39.6 kJ mol^–1^), whereas the corresponding entropy factor is unfavorable (Δ*S* = −46.6 kJ mol^–1^K^–1^). In accordance with the results, the binding of the clathrochelate **3** to BSA macromolecule is driven by the hydrogen bonding and/or van der Waals interactions, while the hydrophobic interactions are not strong enough to overcome the corresponding decrease in a conformational entropy. It should be mentioned that according to the results of fluorescent titration (vide supra), the binding constant is two orders of magnitude higher compared to ITC-obtained data and multiple binding sites could be suggested. The possible explanation may be related with the different detection mechanisms of the two methods, which detect binding with different sites. While the binding with zero enthalpy (i.e., purely entropy-driven) is not detected by ITC, the other which does not affect tryptophan could not be detected by protein fluorescence quenching.

### 3.5. Molecular Modelling Calculations of the Clathrochelate–Albumin Assemblies

**Figure 6:**
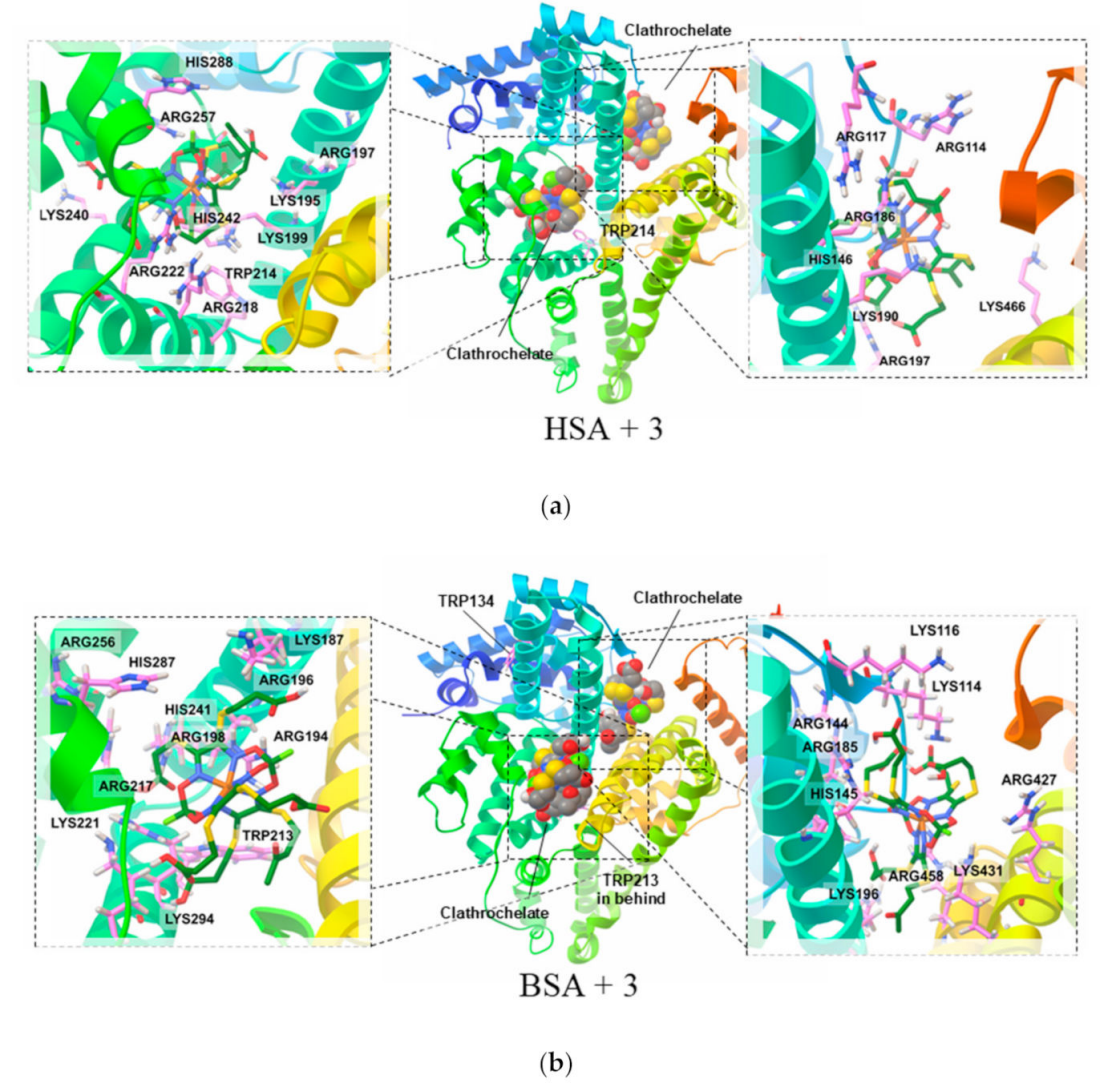
The calculated molecular models of the clathrochelate **3**—HSA [human serum albumin; (**a**) and clathrochelate **3**—BSA (bovine serum albumin; (**b**) guest—host assemblies: their general views (central pictures) and the zoomed views of **3** included in Site I (left side) and in an “additional site” (right side) of these macromolecules. The easily available amino acid residues with charged sidechains and the Trp213/Trp214 residues of their Site I are also shown.

Finally, we performed molecular simulation (by AutoDock Vina program) of the association of BSA and HSA macromolecules with clathrochelate molecule **3**, thus aiming to evaluate a possible structural arrangement of clathrochelate-albumin supramolecular complexes. The results of the docking of **3** to the surface of both HSA and BSA macromolecules indicate that their Site I (Figure 1a,c) is the main site for binding (Figure 6a,b; left side). In addition, interaction of clathrochelate molecule **3** with an “additional site” (Figure 6a,b; right side) seems to be likely as well according to the molecular docking results. At the same time, its binding to Site II (Figure 1b,d) was calculated to have an extremely low energy. Thus, the performed calculations of the docking free energy for the clathrochelate **3** binding to HSA allowed us to obtain an empirical free energy potential of its binding to Site I close to −35 kJ/mol, and for an “additional site” of approximately −31 kJ/mol, while for Site II, this value was as low as −18 kJ/mol. Similar values of energy were also calculated for clathrochelate **3** binding to BSA (−33, −30 and −15 kJ/mol).

The performed visual examination of the obtained conformations of these clathrochelate–protein associates suggests that, indeed, the size and the shape of the cavity of Site I, as well as those of an “additional site”, are in good agreement with the geometrical parameters of the clathrochelate guest molecule **3**. Moreover, Site I and the “additional site” of HSA macromolecule are populated by seven and six Lys and Arg amino acid residues, respectively (Figure 6a), which bear positively charged groups that are complimentary to carboxyl groups of clathrochelates. As for BSA, it bears eight positively charged groups in both Site I and the “additional site” (Figure 6b).

Site II has a much flatter shape, and therefore, its available contact surface for inclusion of molecule **3** is substantially lower. Note that HSA contains five positively charged amino acid residues, while BSA has only four.

The described difference in arrangement of HSA and BSA binding sites is considered to be responsible for the distinct layout of clathrochelate molecules in their assemblies with proteins, which leads to different intensities of induced CD signals.

Finally, it should be mentioned that redocking by AutoDock 4.2 (see SI) showed similar results for BSA, while for HSA clathrochelate **3** preferred Site II over Site I. Still, in the case of HSA, global search favored the same aforementioned possible binding sites (Site I, Site II and “additional site”), though in a different order.

## 4. Conclusions

The ability to induce a strong CD response upon their supramolecular binding to globular proteins was discovered for iron(II) clathrochelates functionalized with one, two or six carboxyalkylsulfide groups. These inherently CD-silent clathrochelates revealed selective optical response upon supramolecular binding to HSA (complexes **1** and **2**), BLG (complex **2**) and BSA (complex **3**). The number of carboxyalkylsulfide substituents per macrobicyclic molecule strongly affects the ICD response in terms of intensity and selectivity. This implies that they form assemblies of different geometry with a different layout of clathrochelate molecules (as it follows from their ICD spectra).

The formation of both 1:1 and 1:2 supramolecular assemblies of HSA with the clathrochelate guests was evidenced by ESI-TOF MS experiments. More interestingly, such clathrochelate-to-protein binding can take place even if the corresponding assembly remains CD-silent (for example in the case of **3**–HSA associate).

The results of the performed protein fluorescence quenching study suggest that **3** binds with both BSA and HSA macromolecules via the sites which are in close proximity with Trp residues. The fluorescence titration experiments allowed us to determine the 1:1 stoichiometry of this associate and the value of the binding constant for **3** to HSA equal to 5.4 × 10^5^ M^–1^. In contrast, in the case of BSA, the fluorescence titration data suggest a multiple binding of the clathrochelate molecules of **3** to the protein.

The binding constant of the 1:1 assembly **3**–BSA that is equal to 3.2 × 10^4^ M^–1^ was determined from ITC measurement, which indicates that this association is an enthalpy-driven process.

Molecular docking calculations revealed that for both BSA and HSA, a supramolecular binding of **3** to Site I, as well as its binding to the “additional site”, are more favorable energetically than binding to Site II.

Comparison of optical characteristics and binding properties of the new carboxyalkylsulfide iron(II) clathrochelates with those of previously described carboxyphenylsulfide analogs indicates a substantial difference in their reporting properties. This may be explained by the possibility of π-stacking (aromatic) interactions of aromatic fragments of the carboxyphenylsulfide with aromatic groups of protein amino acids. Additionally, this difference could be also caused by a higher structural flexibility of the carboxyalkylsulfide chains in new complexes when compared to that of carboxyphenylsulfide groups in previously reported clathrochelates.

## Figures and Tables

**Table 1:** Coordinates of the binding sites in human (HSA) and bovine (BSA) serum albumin structures used for the docking calculations, and their RCSB IDs.

	HSA (RCSB ID: 4L8U)			BSA ((RCSB ID: 4JK4)		
	**x**	**y**	**z**	**x**	**y**	**z**
Site I	28.8	15.2	17.6	95.9	18.5	20.7
Site II	3.7	−3.8	6.9	112	40	37

**Table 2:** Intensities of induced circular dichroism signals (ΔICD; mdeg) for the iron(II) clathrochelates **1**–**3** induced in the presence of globular proteins bovine (BSA) and human (HSA) serum albumin, lysozyme (LYZ) and beta-lactoglobulin (BLG) (c_protein_ = 4 × 10^–5^ M, c_clt_ = 2 × 10^–5^ M) in 0.05 M Tris–HCl buffer with pH 7.9.

Complex	+BSA	+HSA	+LYZ	+BLG
**1**	3.0	9.8	1.0	1.5
**2**	2.5	20.8	1.1	18.5
**3**	24.9	0.9	0.8	0.5

**Table 3:** Averaged deconvoluted masses (Da) of the detected charge states (+15 to +19) of human serum albumin (HSA; delipidated) and its adducts with clathrochelates **1**–**3**. The mass differences of the adducts with respect to unreacted HSA are given, as well as theoretical masses of clathrochelates.

Species	Deconvoluted Mass [Da]	Mass Difference to Reference [Da]	Theoretical Mass of Compound [Da]
Delipidated HSA	66,802 ± 21		
+**1**	67,721 ± 34	919	782
+2× **1**	68,328 ± 32	2 × 763	2 × 782
+**2**	67,800 ± 29	998	886
+2× **2**	68,573 ± 36	2 × 885	2 × 886
+**3**	68,052 ± 58	1250	1075
+2× **3**	69,085 ± 64	2 × 1142	2 × 1075

## Supplementary Materials

The following are available online at https://www.mdpi.com/2218-273X/10/12/1602/s1.
