# Supplementary material for: Sensing of Proteins by ICD Response of Iron(II) Clathrochelates Functionalized by Carboxyalkylsulfide Groups
Source: Biomolecules. 2020 Nov 26;10(12):1602. doi: 10.3390/biom10121602 (PMC7759900; doi:10.3390/biom10121602)
Supplement: Supplementary file 1 [file biomolecules-10-01602-s001.zip › Supporting Information.docx]

**Supporting Information to:**

Sensing of proteins by ICD response of iron(II) clathrochelates functionalized by carboxyalkylsulfide groups

Mykhaylo Losytskyy ^1,^*, Nina Chornenka ^2^, Serhii Vakarov ^2^, Samuel M. Meier-Menches ^3^, Cristopher Gerner ^3^, Slawomir Potocki ^4^, Vladimir B. Arion ^5^, Elzbieta Gumienna-Kontecka ^4^, Yan Voloshin ^6,7^ and Vladyslava Kovalska

^1^ Institute of Molecular Biology and Genetics NASU, 150 Zabolotnogo St., 03143 Kyiv, Ukraine; [v.kovalska@gmail.com](mailto:v.kovalska@gmail.com) (V.K.)

^2^ Vernadsky Institute of General and Inorganic Chemistry NASU, 32/34 Palladina Av., 03142 Kyiv, Ukraine; [nina.v.chornenka@gmail.com](mailto:nina.v.chornenka@gmail.com) (N.C.); [vakarov.s.v@gmail.com](mailto:vakarov.s.v@gmail.com) (S.V.)

^3^ Institute of Analytical Chemistry, University of Vienna, Währinger Strasse, 38, A-1090, Vienna, Austria; [samuel.meier@univie.ac.at](mailto:samuel.meier@univie.ac.at) (S.M-M.); [christopher.gerner@univie.ac.at](mailto:christopher.gerner@univie.ac.at) (C.G.)

^4^ Faculty of Chemistry, University of Wroclaw, 14 F. Joliot-Curie St., 50-383 Wroclaw, Poland; [slawomir.potocki@chem.uni.wroc.pl](mailto:slawomir.potocki@chem.uni.wroc.pl) (S.P.); [elzbieta.gumienna-kontecka@chem.uni.wroc.pl](mailto:elzbieta.gumienna-kontecka@chem.uni.wroc.pl) (E.G-K.)

^5^ Institute of Inorganic Chemistry, University of Vienna, Währinger Strasse, 42, A-1090, Vienna, Austria; [vladimir.arion@univie.ac.at](mailto:vladimir.arion@univie.ac.at) (V.A.)

^6^ Nesmeyanov Institute of Organoelement Compounds RAS, 28 Vavilova St., 119991, Moscow, Russia; [voloshin@ineos.ac.ru](mailto:voloshin@ineos.ac.ru) (Y.V)

^7^ Kurnakov Institute of General and Inorganic Chemistry RAS, 31 Leninsky prosp., 119991 Moscow, Russia

***** Correspondence: Correspondence: mlosytskyy@gmail.com (M.L.)

**Redocking in AutoDock 4.2:**

For the comparison with the results obtained by docking in AutoDock Vina, we have also performed redocking in AutoDock 4.2.

The following charges for ligands were used (see Table):

a) Muller charges from calculations, and

b) Gasteiger charges were added to optimized structure.

Boron atoms were then manually changed to C in pdbqt file as AutoDock 4.2 doesn’t have parameters for it while keeping charges.

Coordinates for sites 1 and 2 were taken from table 1 in the main article.

For BSA, AutoDock 4.2 gives the results similar to those obtained by AutoDock Vina. Conformations differ slightly but are in proximity to the same positively charged residues as described in the article. Global search favours Site 1 and “additional site”

For HSA, AutoDock 4.2 results differs from AutoDock Vina results. Global search prefers Site 2 and “additional site” as on the figure 6, a (right side) in the main article. In this case, we believe that the results of AutoDock Vina are more reliable than these of AutoDock 4.2 [1–3].

*Table. Charges for ligands, used for redocking in* AutoDock *4.2.*

| Protein | BSA | | | HSA | |
| --- | --- | --- | --- | --- | --- |
| Structure | 4jk4 | | | 4L8U | |
| Ligand form | Deprotonated | Deprotonated | Protonated | Deprotonated | Deprotonated |
| Charges | Gasteiger | Mueller | Gasteiger | Gasteiger | Mueller |
| Site 1 | -2.02 | -4.56 | -0.39 | -1.23 | -0.51 |
| Site 2 | -0.32 | -1.55 | +3.58 | -2.26 | -1.75 |

**References**

1. M. Chang, C. Ayeni, S. Breuer, B. Torbett. Virtual Screening for HIV Protease Inhibitors: A Comparison of AutoDock 4 and Vina. *PLoS ONE*, 2010, 5(8), e11955.

2. Nguyen Thanh Nguyen, Trung Hai Nguyen, T. Ngoc Han Pham, Nguyen Truong Huy, Mai Van Bay, Minh Quan Pham, Pham Cam Nam, Van V. Vu, and Son Tung Ngo. Autodock Vina Adopts More Accurate Binding Poses but Autodock4 Forms Better Binding Affinity. *Journal of Chemical Information and Modeling*, 2020, 60 (1), 204-211. DOI: 10.1021/acs.jcim.9b00778

3. Oleg Trott and Arthur J. Olson. AutoDock Vina: improving the speed and accuracy of docking with a new scoring function, efficient optimization and multithreading. *J. Comput. Chem*., 2010, 31(2): 455-461. Doi: 10.1002/jcc.21334
